# Combination of ultrasonography and MRI for preoperative prediction of lymph node metastasis in tongue squamous cell carcinoma: An exploratory study

**DOI:** 10.1371/journal.pone.0340884

**Published:** 2026-01-16

**Authors:** Hiroshi Hijioka, Hiroaki Tabata

**Affiliations:** 1 Department of Maxillofacial Diagnostic and Surgical Science, Field of Oral and Maxillofacial Rehabilitation, Graduate School of Medical and Dental Science, Kagoshima University, Kagoshima, Japan; 2 Tabata Dental Clinic, Kagoshima, Japan; All India Institute of Medical Sciences, INDIA

## Abstract

Preoperative depth of invasion (DOI) is a critical predictor of cervical lymph node metastasis (CLNM) in tongue squamous cell carcinoma (TSCC). Ultrasonography (US) offers high accuracy for shallow tumors, whereas magnetic resonance imaging (MRI) provides superior visualization of deeper structures. However, each modality has limitations. This retrospective, exploratory study aimed to investigate a combined strategy leveraging the complementary strengths of both modalities to improve preoperative CLNM prediction. The study included 46 patients with TSCC who underwent radical surgery between September 2014 and August 2019. Correlations between US-derived DOI (usDOI), MRI-derived DOI (mrDOI), and pathological DOI (pDOI) were assessed using Pearson’s product-moment correlation. Agreement was evaluated by Bland–Altman analysis. A grid search approach identified the optimal usDOI threshold for switching to mrDOI, and predictive performance for CLNM was evaluated using receiver operating characteristic (ROC) analysis with the area under the curve (AUC). Both usDOI and mrDOI showed strong correlations with pDOI (r = 0.956 and r = 0.958, respectively). However, usDOI demonstrated smaller measurement bias (mean difference: 0.88 mm) compared to mrDOI (2.43 mm). Bland–Altman plots revealed that usDOI tended to underestimate pDOI when the mean DOI exceeded 10 mm. Grid search analysis determined an optimal threshold of 7 mm for switching from US to MRI measurements. The combined DOI strategy —using usDOI for DOI ≤ 7 mm and mrDOI for DOI > 7 mm— yielded a numerically higher AUC (0.694) compared to either modality alone (usDOI: AUC = 0.682; mrDOI: AUC = 0.673). This study demonstrates the potential value of a conditional imaging strategy that applies US for shallow lesions and MRI for deeper ones. By capitalizing on the strengths of each modality, this approach offers a logical framework for improving the accuracy of preoperative CLNM prediction in TSCC. Larger, prospective studies are warranted to validate these findings.

## Introduction

Squamous cell carcinoma (SCC) is the most common malignancy of the oral cavity, and the tongue is the most affected site. The 5-year survival rate for patients with TSCC has not improved over the last three decades and remains as low as 65% [1]. Cervical lymph node metastasis (CLNM) is the most important prognostic factor for TSCC, and the 5-year survival rate reportedly decreases with lymph node metastases [2,3]. Accurate preoperative prediction of CLNM is crucial for optimizing treatment strategies and preserving post-treatment quality of life.

The 8th edition of the Union for International Cancer Control (UICC) TNM classification added depth of invasion (DOI) to the T-category of the TNM classification for oral cancer because DOI is strongly associated with CLNM. The National Comprehensive Cancer Network (NCCN) guidelines have also set a DOI of 4 mm as the cutoff value for elective neck dissection (END). However, the NCCN-defined DOI is a pathological finding (pDOI), making its preoperative evaluation a significant clinical challenge. Consequently, accurate preoperative DOI assessment via diagnostic imaging is essential. Despite this need, uniform criteria for imaging modalities and measurement techniques have not been established. Although some studies used magnetic resonance imaging (MRI) to determine tumor thickness, there are only a few radiological reports with respect to DOI [4–6]. Radiological criteria must be established for reproducible and accurate assessment of the DOI because substantial intra- and inter-rater variations in radiological assessment cannot be eliminated even amongst experienced readers [7,8]. Studies that attempted to predict CLNM using ultrasonography (US) or MRI did not find a significant cutoff value for the DOI [9–11].

The primary objective of this exploratory study was to investigate a novel strategy for improving the preoperative prediction of CLNM by leveraging the complementary strengths of US and MRI. Recognizing that US offers high accuracy for shallower tumors while MRI provides a comprehensive view of deeper structures, this study sought to explore how these modalities could be combined. It therefore investigated, via a grid search approach, an optimal method for integrating usDOI and mrDOI to enhance predictive accuracy for CLNM, aiming to establish a foundation for future, more definitive studies.

## Materials and methods

### Patients

The characteristics of the forty-six patients with TSCC who underwent radical surgery at the Department of Oral Surgery in Kagoshima University Hospital between September 2014 and August 2019 are presented in Table 1. Exclusion criteria were patients who had received chemotherapy and/or radiotherapy prior to surgery. Patients underwent US and/or MRI examinations suitable for DOI evaluation before histological examination. Of the 46 patients, those for whom measurements were available for each modality (US: 24, MRI: 44) were included in this analysis. The primary analysis of the integrated strategy was conducted on 22 patients for whom complete and valid data were available for all three modalities: US, MRI, and pathology. CLNM was defined as pathological lymph node metastasis found in primary (n = 10) and delayed (n = 10) neck dissection, for a total of 20 cases with CLNM. Baseline patient characteristics are presented using clinical staging (cTNM) to reflect the preoperative clinical scenario. Pathological staging (pTNM) for the analyzed subgroups is provided in the Supporting Information (S1 Table).

**Table 1:** Patient characteristics. The table below details the demographic and clinical characteristics of the 46 patients included in the study, categorized by total cohort and CLNM status.

Characteristic	Category	Total (N = 46)	CLNM Negative (n = 26)	CLNM Positive (n = 20)
Age (years)	Median	72 [62-77]	71 [65-75]	75.5 [61-81]
Sex	Male, n (%)	30 (65.2)	18 (69.2)	12 (60.0)
	Female, n (%)	16 (34.8)	8 (30.8)	8 (40.0)
cT	T1, n (%)	8 (17.4)	6 (23.1)	2 (10.0)
	T2, n (%)	16 (34.8)	11 (42.3)	5 (25.0)
	T3, n (%)	16 (34.8)	6 (23.1)	10 (50.0)
	T4, n (%)	6 (13.0)	3 (11.5)	3 (15.0)
cN	N0, n (%)	31 (67.4)	19 (73.1)	12 (60.0)
	N1, n (%)	5 (10.9)	3 (11.5)	2 (10.0)
	N2, n (%)	8 (17.4)	3 (11.5)	5 (25.0)
	N3, n (%)	2 (4.3)	1 (3.8)	1 (5.0)
cStage	Ⅰ, n (%)	8 (17.4)	6 (23.1)	2 (10.0)
	Ⅱ, n (%)	13 (28.3)	7 (26.9)	6 (30.0)
	Ⅲ, n (%)	13 (28.3)	8 (30.8)	5 (25.0)
	Ⅳ, n (%)	12 (26.1)	5 (19.2)	7 (35.0)

Abbreviations: CLNM, cervical lymph node metastasis.

This retrospective study was approved by Ethics Committee on Epidemiological and its related Studies, Sakuragaoka Campus, Kagoshima University (approval number: 200117).

The requirement for informed consent was waived by the ethics committee. The patient records used in this study were accessed between January 20^th^ 2022 and August 15^th^ 2025. The authors had access to information that could identify individual participants during and after data collection.

### Evaluation of DOI using US and MRI

**Fig 1:**
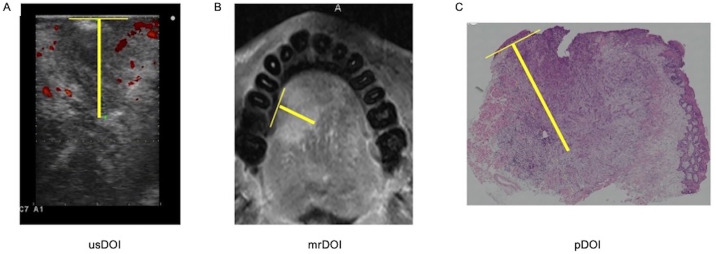
How DOI is measured. Distance from the virtual normal basal mucosa distance to the deepest part of the tumor by ultrasonography (A) and magnetic resonance imaging (B). Distance from the basement of the virtual normal mucosal epithelium to the deepest part of the tumor by pathology (C). usDOI: depth of invasion by ultrasound, mrDOI: depth of invasion by magnetic resonance imaging, pDOI: pathological depth of invasion.

The DOI was determined using US (usDOI) by placing the probe on the tumor, either on the dorsal side of the tongue or perpendicular to the tumor, to observe the lesion. The usDOI measurements were performed using B-mode and color Doppler US images from a ProSound α7 (Hitachi, Tokyo, Japan) fitted with a hockey stick-shaped probe after using nonsterile water-soluble ultrasound gel as an acoustic coupler. The transducer was thinly coated with gel and covered with a plastic wrap. The “virtual normal mucosal base” for usDOI measurement was based on the border between the mucosa/submucosa and the muscularis propria of the tongue in the normal tissue adjacent to the tumor, and this was extrapolated as a line crossing the tumor base. The extent of cancer invasion was determined as the border of the abnormal echo area, and the deepest point was determined as the maximum vertical distance from this extrapolated reference plane. This measurement was clearly distinguished from the overall tumor thickness (Fig 1A).

The DOI was determined by performing MRI (mrDOI) of the normal mucosa adjacent to the deepest aspect of the tumor. MAGNETOM AERA 1.5 T (Siemens Healthineers, Erlangen, Germany) or Achieva 3.0 T TX (Philips Medical Systems, Best, The Netherlands) was used for MRI. The mrDOI was measured from the extrapolation line of the normal mucosal surface to the maximum tumor extension point using T1-weighted images after contrast enhancement with fat suppression (T1WI+C FS). T2-weighted images were used as secondary references (Fig 1B). All DOI measurements were performed by a single author (H.T.). To ensure consistency and minimize measurement bias, a subset of images was re-evaluated by the same author at a two-week interval, confirming high intra-rater reliability.

### Pathological evaluation of DOI

Tissue samples were obtained from specimens excised along the coronal plane for histopathological analysis. They were fixed in 10% neutral buffered formalin, embedded, sectioned, and stained with hematoxylin and eosin. The pDOI was measured according to the 8th edition of the AJCC cancer staging manual, which states that the DOI is measured by first finding the horizon of the basement membrane of the adjacent normal squamous mucosa, followed by establishing a perpendicular plumb line from this horizon to the deepest point of tumor invasion (Fig 1C).

### Statistical analysis

Continuous variables were summarized as medians with interquartile ranges (IQRs), and categorical variables as frequencies and percentages; the χ² test was used for group comparisons. Correlations between imaging-based DOI (usDOI, mrDOI) and pathological DOI (pDOI) were evaluated using Pearson’s product-moment correlation and simple linear regression analysis, while their agreement was assessed by Bland-Altman analysis. For the primary objective of creating a composite indicator (us/mrDOI), a data-driven grid search was implemented to identify the optimal threshold for switching from usDOI to mrDOI in predicting CLNM. The predictive performance of this composite strategy was evaluated using logistic regression and receiver operating characteristic (ROC) curve analysis, with the area under the curve (AUC) serving as the primary metric. A two-sided P-value < 0.05 was considered statistically significant. All statistical analyses were performed in R (version 4.4.2, R Foundation for Statistical Computing, Vienna, Austria) and JMP Pro 18 (SAS Institute, Cary, NC, USA).

To determine the optimal threshold for switching from usDOI to mrDOI, a grid search analysis was performed. Instead of fixed increments, candidate thresholds were defined based on the 10th to 90th percentiles of the usDOI distribution in the analyzed cohort (n = 22). Specifically, the following nine values were tested: 4.14, 4.70, 5.00, 5.52, 7.00, 8.00, 9.38, 10.56, and 14.78 mm. For each candidate threshold, a composite DOI variable was generated (replacing usDOI with mrDOI when usDOI > threshold), and a logistic regression model for predicting CLNM was fitted. The threshold that yielded the maximum Area Under the Curve (AUC) was identified as the optimal cutoff point.

## Results

### Correlations between usDOI/mrDOI and pDOI

**Fig 2:**
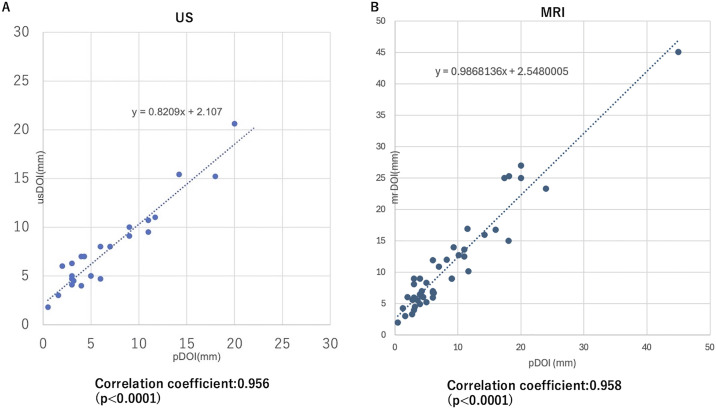
Correlation between imaging-derived and pathological DOI. **(A)** Correlation between usDOI and pDOI. **(B)** Correlation between mrDOI and pDOI. usDOI: depth of invasion by ultrasound, mrDOI: depth of invasion by magnetic resonance imaging, pDOI: pathological depth of invasion.

Both usDOI and mrDOI demonstrated strong, statistically significant positive correlations with pDOI. A strong positive correlation was found between usDOI and pDOI, with a correlation coefficient of r(22) = 0.956 (p < 0.0001). The mrDOI and pDOI were also strongly correlated, with a correlation coefficient of r(42) = 0.958 (p < 0.0001) (Fig 2).

### Associations between usDOI/mrDOI/pDOI and CLNM

**Fig 3:**
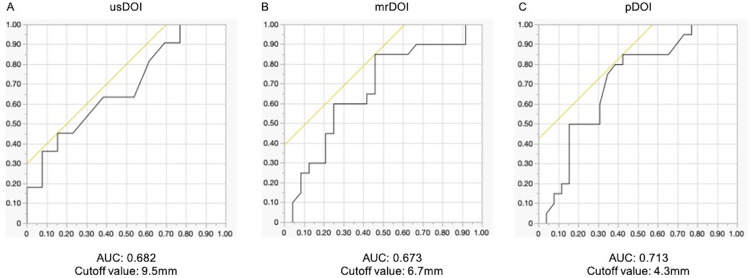
ROC curve analysis for CLNM prediction in individual modality cohorts. **(A)** Association of usDOI and CLNM (n = 24). **(B)** Association of mrDOI and CLNM (n = 44). **(C)** Association of pDOI and CLNM (n = 46). Note: Sample sizes differ between modalities due to data availability. usDOI: depth of invasion by ultrasound, mrDOI: depth of invasion by magnetic resonance imaging, pDOI: pathological depth of invasion.

ROC curves were generated for predicting CLNM using usDOI, mrDOI, and pDOI. The AUC and optimal cutoff values were as follows: usDOI: AUC = 0.682, cutoff = 9.5 mm; mrDOI: AUC = 0.673, cutoff = 6.7 mm; pDOI: AUC = 0.713, cutoff = 4.3 mm (Fig 3).

### Agreement between usDOI/mrDOI and pDOI

**Fig 4:**
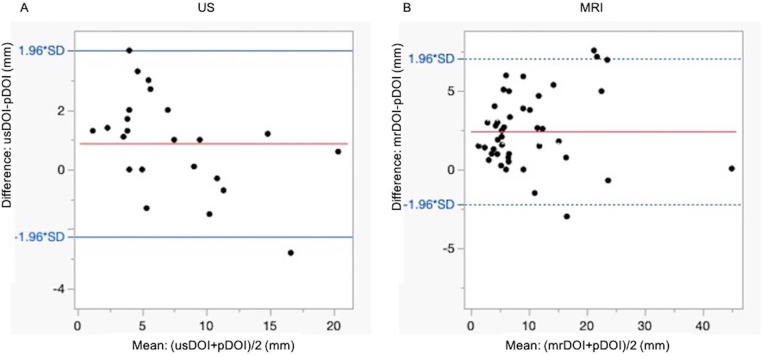
Bland–Altman analysis of agreement between imaging and pathological DOI. **(A)** Agreement between usDOI and pDOI (mm). **(B)** Agreement between mrDOI and pDOI (mm). The plots illustrate the difference between usDOI or mrDOI and pDOI against their mean values. The solid red line represents the mean difference (bias), while the dashed blue lines indicate the 95% limits of agreement (mean±1.96 × SD).

The agreement between usDOI/mrDOI and pDOI was evaluated using Bland-Altman analysis. The mean difference between usDOI and pDOI was 0.88 mm with a standard deviation of 1.59 mm. The mean difference between mrDOI and pDOI was 2.43 mm with a standard deviation of 2.39 mm. In the comparison between usDOI and pDOI, usDOI tended to underestimate pDOI when the mean DOI exceeded 10 mm. In contrast, the comparison between mrDOI and pDOI showed a larger measurement bias and greater variability overall (Fig 4).

### Prediction of CLNM by the combination of usDOI and mrDOI (us/mrDOI)

**Fig 5:**
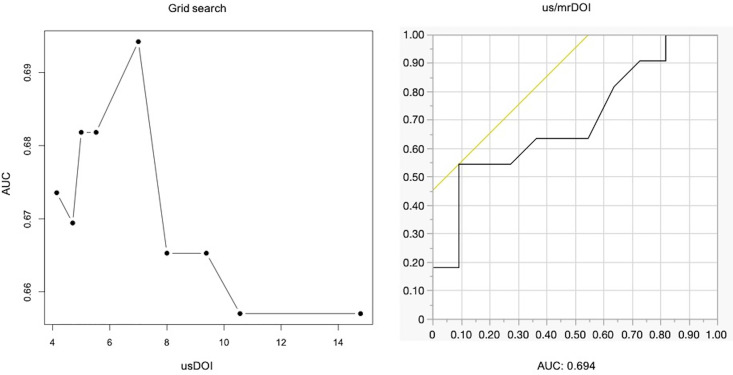
Diagnostic performance of the combined us/mrDOI strategy for predicting cervical lymph node metastasis. **(A)** Grid search analysis showing the relationship between usDOI thresholds (mm) and the corresponding area under the ROC curve (AUC). The highest AUC was observed at a usDOI threshold of 7 mm. **(B)** Receiver operating characteristic (ROC) curve for the combined us/mrDOI strategy (n = 22), with an AUC of 0.694. Note: The cohort for this analysis consists only of patients with complete data for all modalities. Direct comparison of AUC values with Fig 3 should be interpreted with caution due to the difference in sample populations.

The results of the Bland-Altman plot showed that usDOI had a smaller mean difference compared to mrDOI, with many cases falling within clinically acceptable limits. However, it tended to be underestimated as the DOI became deeper. Furthermore, the variability of mrDOI was larger than that of usDOI even in cases with small DOI. Based on these complementary characteristics, a composite indicator (us/mrDOI) was established, referring to usDOI for shallower depths and mrDOI for depths exceeding a specific threshold. This threshold was comprehensively explored from usDOI values using a grid search with AUC as the evaluation metric. As a result, the optimal cutoff value was identified as 7 mm, with a corresponding AUC of 0.694. The us/mrDOI constructed using this optimal cutoff demonstrated higher predictive ability for CLNM compared to usDOI alone or mrDOI alone (Fig 5).

## Discussion

TSCC is characterized by a great propensity for CLNM, which affects the probability of regional control and survival [12]. The detection of metastatic neck disease at the time of initial treatment for SCC is a critical goal of imaging modalities. The need for elective neck dissection in patients with N0 neck is often based on clinical and/or radiographic assessment of tumor depth of invasion in addition to tumor size [10]. Computed tomography (CT), MRI, and US are currently used for preoperative assessment of the primary tumor and cervical node status. The assessment of CLNM using CT, MRI, and US is based on morphological imaging parameters, such as nodal size, shape, and contrast-enhancement pattern, but not on metabolic activity. A previous study by the author (H.H.) established chart-assessment criteria based on integrated imaging results, which improved the assessment of delayed CLNM but was not sufficiently regulated [13]. This suggests inherent limitations in imaging techniques that focus exclusively on lymph nodes rather than primary tumor characteristics.

Several previous studies have reported a correlation between delayed CLNM and tumor thickness [6,14,15]. The DOI (specifically pDOI) has been reported to be a major predictor and prognostic determinant of CLNM [5,16–19]. The pDOI is difficult to confirm preoperatively because it is measured using formalin-fixed pathological specimens. Imaging examinations are generally recommended for accurate preoperative evaluation of the DOI. The DOI is significantly associated with regional metastasis and has recently been included as an important factor in the T-categorization of SCC in the 8th edition of the AJCC cancer staging manual. However, the staging manual only provides the method for measuring pDOI and does not specify the method for preoperative usDOI, mrDOI measurement, which requires standardization [20]. Determining the most effective imaging technique is one of the most important issues affecting CLNM prediction with DOI. The cutoff value of the DOI for determining prognosis is most commonly set at 4 mm [17,21,22]. Huang et al. previously reported that a DOI exceeding 4 mm was a strong predictor of CLNM [21]. Some studies have reported the clinical utility of US and MRI in determining the DOI of TSCC [4,5,9,10,13,23,24]. Several studies have reported that radiological tumor thickness measured on contrast-enhanced T1-weighted imaging significantly correlated with histological tumor thickness [4,5,24]. A correlation between mrDOI and pDOI (correlation coefficient, 0.68; p < 0.0001) has also been reported [9]. A systematic review evaluating the relationship between MRI and DOI reported that compared with pDOI, mrDOI showed a statistically significant overestimation of 1.90 mm, with 1.5 Tesla devices being superior to 3.0 Tesla devices [25]. Baek et al. recently confirmed the utility of intraoral US in the prediction of pathologic tumor thickness of TSCC [24]. Moreover, they underlined the limitations of both CT and MRI for the evaluation of TSCC, although MRI provided superior information regarding soft tissues compared to that provided by CT. Moreover, another study demonstrated the value of CT in DOI evaluation [15].

One disadvantage of MRI is that it is contraindicated in patients with claustrophobia, non-compliant patients, or those with metal prostheses or pacemakers. Artifacts from metal implants and other prostheses may also affect the accuracy of mrDOI.

By contrast, US is faster, less invasive, and does not require the same degree of patient compliance as MRI. Its higher resolution depicts the DOI with better definition in early-stage TSCC. US also helps to define the extent of the tumor and DOI. A very strong correlation has been reported between usDOI and pDOI (correlation coefficient, 0.87–0.98; p < 0.0001) [21]. US is the most useful method for determining the need for END in patients with SCC, but there are some limitations for the evaluation of usDOI [21,22]. US is an operator-dependent procedure. Moreover, it is difficult to examine tumors located in the posterior oral cavity with US. Perpendicular US evaluation of SCCs located in the posterior third of the tongue is not feasible due to the difficulty in gaining access to this area. The probe should be kept in tight contact with the organ to be evaluated during intraoral US to improve the interface between the probe and tissue, but without exerting too much pressure that could deform or modify the shape of the tumor itself. Lam et al. stated that intraoral US can be used to measure tumor thickness but not depth [5], although some studies assessed tumor depth instead of tumor thickness [26,27]. Hayashi et al. reported that acoustic coupling media is useful for usDOI measurement [28]. Noorlag et al. reported poor accuracy of the preoperative usDOI measurement for determining tumor thickness of pT3 tumors (8th TNM classification), which led to the underestimation of the DOI, while the accuracy of MRI was independent of tumor thickness [29].

The present study found a significant correlation between pDOI and us/mrDOI. Consistent with another study, the individual predictive capacities of usDOI and mrDOI for CLNM were modest in our cohort. [21]. The discrepancy between us/mrDOI and pDOI could be attributed to the shrinkage of the specimen during formalin fixation or inflammatory changes around the tumor, and the tendency of overestimation cannot be ignored. At our institution, the clinical protocol for the majority of patients involved comprehensive imaging (US, MRI, and CT) prior to biopsy. This approach demonstrated a good correlation with pDOI and identified a pDOI cutoff of 4.3 mm for CLNM, which is consistent with the 4 mm NCCN guideline threshold. Iida et al. also reported that a combination of clinical palpation, MRI if necessary, and US may be helpful in pretreatment staging and prognosis, and may provide additional guidance for therapy [30]. Kaltoft et al. prospectively evaluated whether ultrasound or MRI could accurately measure DOI and concluded that although both US and MRI were significantly correlated with DOI, US had a higher correlation coefficient and allowed for more accurate assessment [31]. This study’s findings concur, as Bland-Altman analysis revealed that usDOI had a smaller measurement bias than mrDOI. In the Bland-Altman analysis, usDOI tended to underestimate pDOI when the mean DOI exceeded 10 mm. In clinical practice, underestimation of DOI may lead to undertreatment, such as the omission of elective neck dissection, potentially resulting in missed lymph node metastasis and worsened prognosis. Therefore, overestimation is generally considered more acceptable than underestimation in terms of clinical safety. Our Bland-Altman analysis revealed distinct error profiles: while usDOI is highly accurate for shallow tumors, it tends to underestimate depth in deeper lesions (>10 mm), posing a risk of undertreatment (e.g., omitting neck dissection). Conversely, mrDOI tends to overestimate depth. Although overestimation may lead to wider margins, it provides a safety buffer against missing occult metastases in deep tumors. Therefore, our strategy employs the more accurate US for shallow lesions (≤ 7 mm) and switches to the ‘safer’ MRI for deeper lesions to minimize the risk of underestimation while maintaining diagnostic precision.

This study highlights the potential of a synergistic approach to DOI assessment, capitalizing on the unique advantages of both US and MRI. The findings affirm that US provides excellent accuracy with minimal measurement bias for shallower lesions, whereas MRI, despite a tendency to overestimate due to peritumoral inflammation, is invaluable for assessing deeper infiltration where US can be limited. The proposed 7 mm usDOI cutoff for switching to mrDOI represents a logical, data-driven strategy to harness the strengths of each modality. While the exploratory nature of this threshold warrants confirmation in larger studies to ensure generalizability, it provides a valuable, concrete hypothesis for future validation. This approach pioneers a path toward a more nuanced and potentially more accurate preoperative staging paradigm.

At present, optimal elective neck treatment for patients with TSCC remains controversial and requires further study. TSCC exhibits more aggressive regional metastasis compared to cancer of other parts of the mouth [32]. Therefore, the refined diagnostic approach proposed in this study may be particularly valuable for evaluating neck status and guiding treatment decisions in this high-risk patient population.

A significant limitation of this study is the potential for selection bias arising from the incomplete datasets for each modality. Specifically, usDOI data was available for only 24 of the 46 patients (S1 Fig). This discrepancy stems from the fact that intraoral ultrasonography is not performed as a routine examination for all TSCC cases at our institution. It is preferentially utilized for tumors presumed to have a shallower DOI, where its high resolution is most advantageous. Conversely, its application is limited for tumors located in the posterior tongue or those with greater depth, where technical constraints related to probe positioning can compromise measurement accuracy. This selective application resulted in a smaller and potentially biased sample for the usDOI analysis, a factor that must be considered when interpreting the findings. This study also has several limitations. Beyond its single-center design, small sample size, and the absence of external validation, a further limitation is that all radiological measurements were performed by a single evaluator. While high intra-rater reliability was confirmed, the absence of an inter-rater reliability analysis limits the generalizability of the measurement consistency. Importantly, a formal conversion model to predict pDOI from usDOI was not developed. Although Bland-Altman analysis was used to assess agreement, this does not replace a predictive formula. Developing such a model through regression analysis or machine learning would enhance the clinical utility of usDOI in preoperative assessment.

Within the context of these limitations, both usDOI and mrDOI exhibited a strong correlation with pDOI; however, the absolute difference from pDOI was notably smaller for usDOI, particularly in early-stage cancers. These findings suggest that, in exploratory analyses, clinical DOI assessment could primarily rely on usDOI values, with mrDOI serving as a supplementary reference when usDOI exceeds a certain threshold. The combination of usDOI and mrDOI showed potential for improving the preoperative prediction of CLNM in patients with TSCC. However, these results should be regarded as hypothesis-generating and require confirmation in larger, multi-center studies before they can be considered for clinical implementation. The limited sample size in this study underscores the need for further research to establish and validate a reliable cutoff value for usDOI when guiding combined modality use.

## Conclusions

This exploratory study demonstrates the promising potential of a combined US and MRI strategy to refine the preoperative prediction of CLNM in TSCC. By leveraging the high accuracy of US for more superficial tumors and the comprehensive view of MRI for deeper ones, this complementary approach may offer a more accurate assessment than either modality alone. The findings presented here provide a strong rationale and a clear direction for future large-scale, multi-center validation studies. While further research is essential to confirm these promising results for clinical implementation, this work represents a positive step toward optimizing treatment planning and improving patient outcomes.

## Supporting information

S1 Table Characteristics of patient subgroups used in analyses. (DOCX)

S1 Fig Flow diagram of the patient selection process. The diagram illustrates the flow of participants through the study, from the initial cohort of 46 patients with tongue squamous cell carcinoma to the final subgroups included in the Ultrasound (US) analysis, Magnetic Resonance Imaging (MRI) analysis, and Grid Search analysis. The chart details the number of patients excluded at each stage and the reasons for exclusion. US, ultrasound; pDOI, pathological depth of invasion; mrDOI, MRI-derived depth of invasion.
(TIFF)
