# Supplementary material for: Combination of ultrasonography and MRI for preoperative prediction of lymph node metastasis in tongue squamous cell carcinoma: An exploratory study
Source: PLoS One. 2026 Jan 16;21(1):e0340884. doi: 10.1371/journal.pone.0340884 (PMC12810809; doi:10.1371/journal.pone.0340884)
Supplement: S1 Table — (DOCX) [file pone.0340884.s001.docx]

S1 Table. Characteristics of patient subgroups used in analyses.

| Characteristic | Category | MRI Analysis (N=44) | US Analysis (N=24) | Grid Search (N=22) |
| --- | --- | --- | --- | --- |
| Age (years) | Median | 72 [62.5-76.5] | 68.5 [57.5-76.5] | 68.5 [59-76] |
| Sex | Male, n (%) | 30 (68.2) | 16 (66.7) | 16 (72.7) |
|  | Female, n (%) | 14 (31.8) | 8 (33.3) | 6 (27.3) |
| pT | T1, n (%) | 8 (18.2) | 6 (25.0) | 5 (22.7) |
|  | T2, n (%) | 23 (52.3) | 13 (54.2) | 12 (54.5) |
|  | T3, n (%) | 7 (15.9) | 3 (12.5) | 3 (13.6) |
|  | T4, n (%) | 6 (13.6) | 2 (8.3) | 2 (9.1) |
| pN | N0, n (%) | 34 (77.3) | 18 (75.0) | 16 (72.7) |
|  | N1, n (%) | 3 (6.8) | 2 (8.3) | 2 (9.1) |
|  | N2, n (%) | 6 (13.7) | 4 (16.7) | 4 (18.2) |
|  | N3, n (%) | 1 (2.3) | 0 (0) | 0 (0) |
| pStage | Ⅰ, n (%) | 8 (18.2) | 6 (25.0) | 5 (22.7) |
|  | Ⅱ, n (%) | 21 (47.7) | 11 (45.8) | 10 (45.5) |
|  | Ⅲ, n (%) | 5 (11.4) | 2 (8.3) | 2 (9.1) |
|  | Ⅳ, n (%) | 10 (22.8) | 5 (20.8) | 5 (22.7) |

Abbreviations: pT, pathological T stage; pN, pathological N stage; pStage, pathological stage.
