# Supplementary figures and images for: Combination of ultrasonography and MRI for preoperative prediction of lymph node metastasis in tongue squamous cell carcinoma: An exploratory study
Source: PLoS One. 2026 Jan 16;21(1):e0340884. doi: 10.1371/journal.pone.0340884 (PMC12810809; doi:10.1371/journal.pone.0340884)

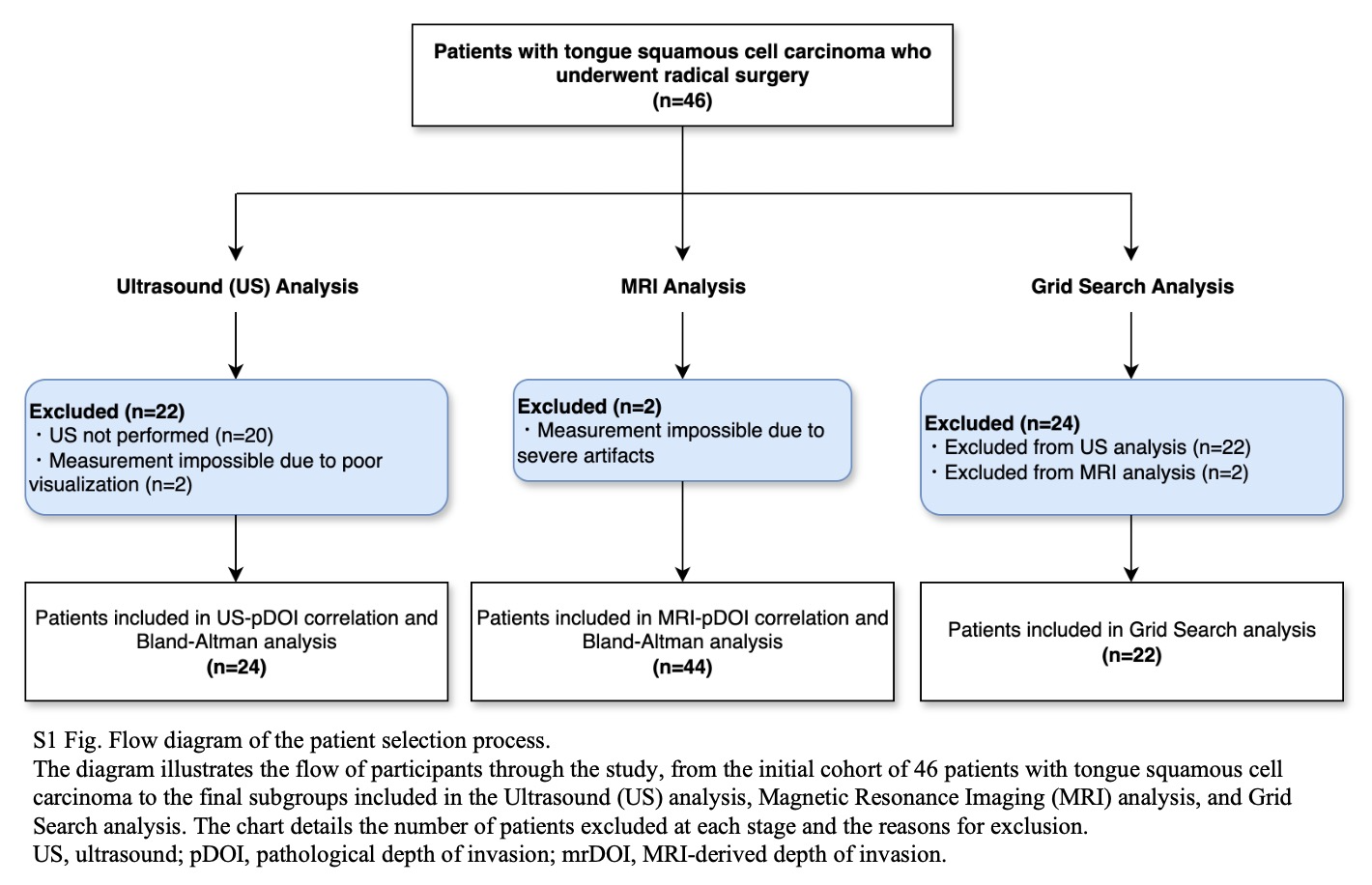

Supplement: S1 Fig — The diagram illustrates the flow of participants through the study, from the initial cohort of 46 patients with tongue squamous cell carcinoma to the final subgroups included in the Ultrasound (US) analysis, Magnetic Resonance Imaging (MRI) analysis, and Grid Search analysis. The chart details the number of patients excluded at each stage and the reasons for exclusion. US, ultrasound; pDOI, pathological depth of invasion; mrDOI, MRI-derived depth of invasion. (TIFF) [file pone.0340884.s002.tiff]
